# Analysis of the culturable endophytic fungi in *Dalbergia odorifera* and screening of the fungi inducing heartwood formation

**DOI:** 10.3389/fpls.2026.1755005

**Published:** 2026-03-31

**Authors:** Hua Dou, Chenlu Fan, Xiaomin Liu, Xuyu Chen, Jianhe Wei

**Affiliations:** 1 Institute of Medicinal Plant Development, Chinese Academy of Medical Sciences & Peking Union Medical College, Beijing, China; 2 Key Laboratory of Resources Conservation and Development of Southern Medicine of Hainan Province, Chinese Academy of Medical Sciences & Peking Union Medical College, Haikou, China; 3 Key Laboratory of State Administration of Traditional Chinese Medicine for Agarwood Sustainable Utilization, Hainan Branch of the Institute of Medicinal Plant Development, Haikou, China

**Keywords:** *Dalbergia odorifera*, endophytic fungi, heartwood formation, *Lasiodiplodia theobromae*, metabolomics

## Abstract

**Introduction:**

This study aimed to investigate the culturable endophytic fungi in the sapwood and heartwood of *Dalbergia odorifera*, to screen fungi that can promote heartwood formation, and analysis of components involved in the formation of heartwood of *D. odorifera* induced by *Lasiodiplodia theobromae.*

**Methods:**

Fungi were isolated by tissue culture method, identified based on morphological characteristics and ITS sequence analysis, and their induction effect was confirmed by thin-layer chromatography. Non-targeted metabolomics was used to analyze the induced heartwood.

**Results:**

A total of 109 endophytic fungal strains were isolated and identified from the six samples. Most isolates (71.56%) came from the heartwood, and other isolates (28.44%) came from the sapwood wood. A strain P3BM2 identified as *L. theobromae*, isolated from the heartwood of *D. odorifera*, has the best effect in inducing the formation of heartwood. Thin-layer chromatography confirmed that the induced heartwood met pharmacopeial standards, and its dimensions exceeded those of the control group. Compared to the control, *L. theobromae* inoculation activated specific differential metabolic pathways.

**Discussion:**

Endophytic fungi are involved in the heartwood formation of *D. odorifera*. In this study, we isolated and identified the culturable endophytic fungi from *D. odorifera*, and demonstrated that *L. theobromae* can promote heartwood formation in this species. This research provides a theoretical basis for the fungal-mediated induction of high-quality heartwood formation in *D. odorifera*.

## 1 Introduction

*Dalbergia odorifera* T. Chen, a member of the Leguminosae family and also known as Jiangxiang, Huanghuali or Hualimu, is a unique endangered precious rosewood species native to Hainan Province of China. It is currently listed as a National Category II protected plant in China. Its dried heartwood from trunks and roots is a well-recognized traditional Chinese medicine recorded in the *Chinese Pharmacopoeia* (2015), with the efficacy of promoting blood circulation, relieving pain and eliminating blood stasis. It is widely used in traditional Chinese medicine prescriptions for cardio-cerebrovascular diseases (He et al., 2022). The chemical composition of *D. odorifera* is complex. Volatile oils and aromatic extracts are its main active components. They are biosynthesized via specific secondary metabolism pathways. These components are clinically used for the early prevention and treatment of chest pain and heartache (He et al., 2022). Due to the gradual decline of wild resources, *D. odorifera* has been introduced and cultivated in southern China. Yet large-scale commercial production remains unachieved to date (He, 2024). This is mainly attributed to the slow and low-yield natural heartwood formation process of this species, which is a tightly regulated molecular and developmental process in woody plants.

The trunk of *D. odorifera* is structurally divided into heartwood and sapwood. Natural heartwood formation is a sophisticated developmental process regulated by a complex network of known endogenous and exogenous regulatory pathways. It includes intrinsic genetic regulation, endogenous phytohormone signaling modulation and exogenous microbial invasion. The endogenous phytohormone signaling modulation mainly involves auxin, cytokinin and abscisic acid (Yang et al., 2025). At the molecular level, this process is driven by the differential expression of a series of functional genes. These genes are associated with cell differentiation, secondary metabolism and stress response. The transformation from sapwood to heartwood relies on a cascade of tightly regulated physiological and biochemical reactions. In this process, secondary metabolism pathways are dramatically activated. This process is accompanied by the alteration of intracellular enzyme activity, the reduction of cell water content, the degradation of storage substances, the directional transport and accumulation of nutrients, and the *de novo* biosynthesis of species-specific secondary metabolites. Typical examples are flavonoids and volatile terpenoids (Yang et al., 2025). The biosynthesis of these secondary metabolites is precisely regulated by key transcription factors and gene expression networks. These secondary metabolites not only define the unique medicinal and commercial properties of high-quality *D. odorifera* heartwood. The properties include purplish-red color, high hardness, oily luster and strong fragrance. They also form the chemical basis of the plant’s defense response to biotic and abiotic stresses. High-quality heartwood endows *D. odorifera* with extremely high economic value in both pharmaceutical and industrial markets, resulting in an enormous demand for high-quality heartwood raw materials (He, 2024).

Plant endophytes are non-pathogenic microorganisms that colonize plant tissues or intercellular spaces for part or all of their life cycle (Kusari et al., 2011). The intricate host-microbe interaction mechanisms between endophytes and host plants are critical for regulating plant growth, development and stress resistance at both physiological and molecular levels. The heartwood and sapwood of *D. odorifera* harbor diverse and abundant endophytic microbial communities. Previous studies have confirmed significant differences in the composition and structure of endophytic fungal and bacterial communities between the two tissues, with higher abundance and diversity detected in the heartwood (Lan et al., 2021; He, 2024). The structural variation of endophytic communities in *D. odorifera* is closely associated with the physical and chemical properties of wood tissues. Among them, pH and water content are the most critical environmental factors regulating the assembly of both endophytic fungal and bacterial communities. Other key factors include ash content, hot-water extractives, and calcium and potassium concentrations (Lan et al., 2021). From a molecular perspective, the assembly of endophytic fungal communities is tightly linked to the activation of host secondary metabolism pathways and defense signaling networks. It forms a dynamic feedback loop between host plant molecular physiology and microbial community structure.

Endophytic microorganisms, especially endophytic fungi, play a pivotal regulatory role in the sapwood-to-heartwood transformation and heartwood formation of *D. odorifera* by modulating the plant’s defense signaling pathways and secondary metabolism networks (He, 2024). Fungal invasion acts as a key biotic stimulus to trigger the host plant’s innate immune defense signaling. It includes the salicylic acid (SA), jasmonic acid (JA) and ethylene (ET) signaling pathways. These pathways are the core of plant defense responses to microbial colonization (Zhao et al., 2020). The activation of these defense signaling pathways further induces the reconstruction of intracellular microenvironment. It also upregulates the expression of key genes in secondary metabolism pathways. The genes are related to flavonoid and terpenoid biosynthesis for instance. This ultimately initiates the physiological and biochemical processes of heartwood formation. The regulatory effect of endophytic fungi on host plants is realized by secreting signaling molecules and effector proteins. These substances can interact with host plant receptors and regulate the expression of downstream functional genes. Accumulating evidence has indicated that specific endophytic fungal taxa are involved in the heartwood formation of *D. odorifera* via these host-microbe interaction mechanisms. Fungal-mediated induction has become a promising strategy for artificial heartwood promotion by regulating the known regulatory pathways of heartwood development (Li et al., 2025). Fungal genera including *Fusarium*, *Pestalotiopsis* and *Hypocrea* have been identified as potential functional taxa. They can modulate plant defense signaling and secondary metabolism at the molecular level to drive sapwood-to-heartwood conversion (Li et al., 2025). Notably, Zhang et al. (2014) reported a Fusarium strain that could effectively induce the formation of pharmacopoeia-compliant heartwood in *D. odorifera* by activating related secondary metabolism pathways and regulating the expression of key functional genes. Jia (2014) further verified the promoting effect of four fungal species on heartwood development via regulating host plant defense and metabolic networks. Elucidating the composition of endophytic fungal communities in the heartwood and sapwood of *D. odorifera* is essential to decipher the molecular and physiological mechanisms underlying fungal-mediated heartwood formation. It is especially important for revealing the crosstalk between host-microbe interactions, plant defense signaling and secondary metabolism pathways at the molecular level. In this study, we focus on culturable endophytic fungi in the heartwood and sapwood of *D. odorifer*a. We aim to identify the core fungal taxa associated with heartwood formation and reveal their regulatory roles in the known heartwood formation pathways from a molecular perspective. This work will lay a solid theoretical foundation for the subsequent screening of functional endophytic fungi and the development of efficient fungal-mediated strategies for high-quality *D. odorifera* heartwood induction by precisely regulating plant defense signaling and secondary metabolism pathways at the molecular level.

## 2 Materials and methods

### 2.1 Sample collection

In May 2024, three 10-year-old trees with comparable trunk diameter and uniform growth that had developed heartwood were selected in a *D. odorifera* plantation baset in Lingao County, Hainan Province, China. It is an area featuring a tropical monsoon climate with an annual average temperature of 23–25 °C and annual precipitation of 1500–1800 mm, where the soil is classified as lateritic soil with pH 4.5-5.5. Stem sections were taken, and the sapwood and heartwood layers were shaved into small pieces using a sterile knife for subsequent experiments. The different sapwood and heartwood samples were labeled BM-1, BM-2, BM-3 and XC-1, XC-2, XC-3, respectively. Because *D. odorifera* is a rare and precious medicinal plant, and wild populations are strictly protected by national policies, and the available cultivated individuals that meet the experimental requirements are limited in the sampling area. The three trees selected in this study were from the same plantation with consistent age, growth status, and environmental conditions such as soil type, rainfall and temperature, which was designed to minimize interference from non-target factors during the initial exploration of endophytic fungal diversity and heartwood induction.

### 2.2 Isolation and identification of endophytic fungi

#### 2.2.1 Isolation of endophytic fungi

Fungi from the six samples were isolated and purified using the tissue separation method. After rinsing the sample surfaces with clean water and slicing them, surface sterilization was performed sequentially with 75% alcohol and 10% sodium hypochlorite solution for 30 seconds each. The samples were then rinsed three times with sterile water to remove residual alcohol and sodium hypochlorite. After drying with sterile filter paper, the tissues were inoculated onto PDA medium supplemented with ampicillin sodium for cultivation, with three tissue pieces per plate arranged in a triangle. Hyphae from the edge of fungal colonies were transferred to new PDA plates and cultured at 28 °C in darkness. After colony growth, hyphae from the colony edges were again transferred to newly prepared PDA plates containing antibiotics for further cultivation. This process was repeated 2–3 times to obtain purified fungal isolates. The efficacy of plant tissue surface sterilization was verified by inoculating the sterile water from the final rinsing step onto PDA medium, followed by incubation and observation of fungal growth to confirm thorough disinfection. The purified fungi were assigned identification numbers and preserved in PDA slant tubes.

#### 2.2.2 Molecular identification of fungi

The purified fungal strains were inoculated into PDB liquid medium. After 7 days, the mycelia were obtained by filtering the culture broth. Fungal genomic DNA was extracted using the Fungal DNA Kit from Guangzhou IGE Biotechnology Co., Ltd. The extracted DNA was used as a template for PCR amplification with Internal Transcribed Spacer (ITS) primers. PCR amplification was performed using ITS1 (5’-TCCGATGGTGAACCTGCGG-3’) and ITS4 (5’-TCCTCCGCTTATTGATATGC-3’) as primers. The PCR reaction mixture (25 μL) contained 1 μL DNA, 1.5 μL each of forward and reverse primer, 12.5 μL of 2 × Taq PCR Master Mix, and 8.5 μL of sterile water. The amplification program was pre-denaturation at 94 °C for 5 min; 30 cycles of denaturation at 94 °C for 1 min, annealing at 50 °C for 1 min, and extension at 72 °C for 1 min; final extension at 72 °C for 7 min; and hold at 4 °C. The PCR products were sequenced. The obtained sequences were compared for homology with sequences in the NCBI GenBank database using BLAST. Prior to phylogenetic tree construction, the optimal nucleotide substitution model was selected via the Model Selection tool in MEGA 11.0 software based on the Bayesian Information Criterion, and the Kimura 2-parameter model with gamma-distributed rate variation among sites (G) was identified as the most suitable. A phylogenetic tree was then constructed using the Neighbor-Joining method in MEGA 11.0, with 1,000 bootstrap replications to assess the reliability of branch nodes.

### 2.3 Validation of heartwood formation induced by endophytic fungi in *D. odorifera*

#### 2.3.1 Validation of fungal promotion of heartwood formation

Purified fungal strains were cultured on PDA medium for 5 days. A 5 mm diameter drill bit was used to create holes angled downward into the stems of *D. odorifera* trees, reaching two-thirds of the stem diameter. Fungal mycelial plugs were inserted into the holes, which were then filled with PDB medium. The drill holes were sealed with tape. Each treatment was performed in triplicate. Control groups included trees with drill holes only (Ctrl-D) and trees with drill holes filled with PDB medium (Ctrl-DPDB).

#### 2.3.2 Identification of the endophytic fungus P3BM2 from *Dalbergia odorifera*

##### 2.3.2.1 Morphological characteristics

A 3mm x 3mm plug of strain P3BM2 was cultured on PDA medium, and its colonial morphology was continuously observed. Temporary slides were prepared, and the mycelial and spore morphology were examined using optical microscopy.

##### 2.3.2.2 Thin-layer chromatography analysis

Six months after fungal inoculation, the *Dalbergia odorifera* trees were felled 30 cm away from the drill holes. The discolored inner regions of the wood were scraped for thin-layer chromatography analysis. The specific method was as follows: 1 g of sample powder was added to 10 ml of methanol, ultrasonicated for 30 minutes, and the supernatant was taken as the test solution. A reference standard sample of *D. odorifera* from the National Institute for Food and Drug Control was prepared into a control solution using the same method. 2 μl of each test solution and control solution were spotted on the same silica gel G plate, using toluene-ethyl acetate (2:1) as the developing solvent. The plate was examined under ultraviolet light (365 nm).

### 2.4 Metabolomic analysis of heartwood formation induced by P3BM2

#### 2.4.1 Sample preparation

Using the *D. odorifera* samples from the heartwood formation validation assay in section 2.3 as the subject of study, 50 mg of the induced product sample was weighed. 1000 μL of extraction solvent containing internal standard (methanol: acetonitrile: water = 2:2:1, internal standard concentration 20 mg/L) was added, followed by vortex mixing for 30 seconds. A grinding instrument with steel beads was used to process the mixture at 45 Hz for 10 minutes, followed by ice-water bath ultrasonication for 10 minutes.

#### 2.4.2 Chromatographic and mass spectrometric conditions

Chromatographic conditions: The LC-MS system consisted of an Acquity I-Class PLUS ultra-performance liquid chromatograph coupled with a Xevo G2-XS QTOF high-resolution mass spectrometer. An Acquity UPLC HSS T3 column (1.8 μm, 2.1 × 100 mm) was used with a flow rate of 0.4 mL/min and an injection volume of 2 μL.

Mass spectrometric conditions: Data were acquired in positive and negative ion modes using an electrospray ionization (ESI) source. Capillary voltage: 2500 V (positive ion mode) or -2000 V (negative ion mode); cone voltage: 30 V; ion source temperature: 100 °C; desolvation gas temperature: 500 °C; cone gas flow rate: 50 L/h; desolvation gas flow rate: 800 L/h; mass-to-charge ratio (m/z) scan range: 50-1200.

#### 2.4.3 Data analysis

After normalizing the original peak area information with the total peak area, the follow-up analysis was performed. Principal component analysis and Spearman correlation analysis were used to judge the repeatability of the samples within the group and the quality control samples. The identified compounds are searched for classification and pathway information in KEGG, HMDB, and lipidmaps databases. According to the grouping information, calculate and compare the difference multiples, A T-test was used to calculate the difference significance *P*-value of each compound. The R language package ropls was used to perform OPLS-DA modeling, and 200 times permutation tests were performed to verify the reliability of the model. The VIP value of the model was calculated using multiple cross-validations. The method of combining the difference multiple, the *P*-value and the VIP value of the OPLS-DA model was adopted to screen the differential metabolites. The screening criteria are FC>1, *P*-value<0.05 and VIP>1. The different metabolites of KEGG pathway enrichment significance were calculated using the hypergeometric distribution test.

## 3 Results

### 3.1 Analysis of endophytic fungal diversity in *D. odorifera* using the tissue isolation method

The tissue isolation method was applied to *D. odorifera* samples for obtaining its endophytic fungi. A total of 109 endophytic fungal strains were isolated and identified from the six samples by the tissue separation method. The distribution of isolates from different parts was shown in **Table 1**. Specifically, 31 and 78 strains were isolated from the sapwood and heartwood layers, accounting for 28.44% and 71.56% of the total isolates, respectively. From the three tree trunks, 43, 26, and 40 endophytic fungal strains were isolated, representing 39.45%, 23.85%, and 36.70% of the total isolates, respectively.

**Table 1:** Number of isolated strains from different samples.

Sample name	Number of isolates	Proportion
BM-1	15	13.76%
XC-1	28	25.69%
BM-2	8	7.34%
XC-2	18	16.51%
BM-3	8	7.34%
XC-3	32	29.36%

**Figure 1:**
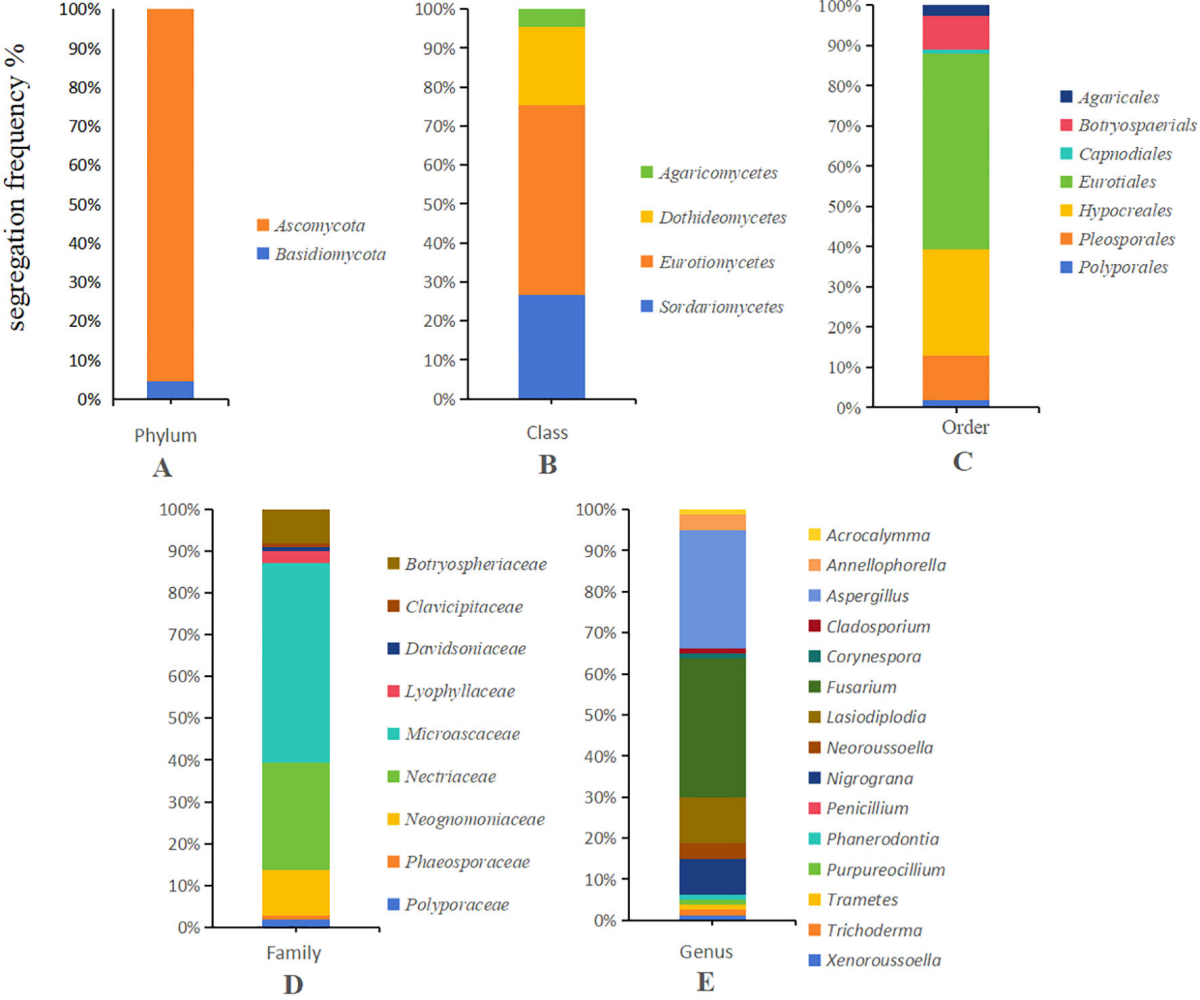
Distribution of endophytic fungi isolated from *D. odorifera* via the tissue separation method at different taxonomic levels. **(A)** Phylum-level classification of fungi, **(B)** Class-level classification of fungi, **(C)** Order-level classification of fungi, **(D)** Family-level classification of fungi, **(E)** Genus-level classification of fungi.

The endophytic fungi cultured on PDA plates were subjected to PCR amplification using ITS1/4 primers, and the resulting sequences were sequenced to obtain corresponding sequence information. The DNA sequences of each strain were compared in NCBI, and the sequence with the highest similarity was selected as the species identification result (**Table 2**). The 109 endophytic strains were classified accordingly. The isolation frequencies of these strains at the genus, family, order, class, and phylum levels are shown in **Figure 1**. Among these, 104 strains belonged to the Ascomycota phylum, and 5 strains belonged to the Basidiomycota phylum. The endophytic fungi isolated from the heartwood layer of *D. odorifera* belonged to 2 phyla, 4 classes, 7 orders, 9 families, 11 genera, and 25 species, demonstrating considerable diversity. Among the isolated strains, *Penicillium citrinum* was the most abundant with 14 strains, followed by *Fusarium solani* with 13 strains, *Aspergillus flavus* with 12 strains, *Lasiodiplodia theobromae* with 9 strains, and *Aspergillus niger* with 6 strains. They were the dominant species, with isolation frequencies of 12.84%, 11.93%, 11.01%, 8.26%, and 5.50%, respectively.

**Table 2:** Sequence identification of endophytic fungi in *D. odorifera*.

NO.	Identification results	Number	Similar sequence from GeneBank Database (Accession number)
1	*Acrocalymma medicaginis*	1	PV248817.1
2	*Annellophorella ellisii*	3	PQ600601.1
3	*Aspergillus aculeatinus*	1	PX443315.1
4	*Aspergillus clavatus*	1	FR733876.1
5	*Aspergillus dimorphicus*	2	PP851743.1
6	*Aspergillus flavus*	12	MT983203.1
7	*Aspergillus niger*	6	NR111348.1
8	*Aspergillus westerdijkiae*	1	KY264741.1
9	*Corynespora cassiicola*	1	MT549027.1
10	*Cladosporium cladosporioides*	1	AM182183.1
11	*Fusarium akasia*	5	MN954354.1
12	*Fusarium ambrosium*	2	PP695544.1
13	*Fusarium avenaceum*	2	KU852623.1
14	*Fusarium decemcellulare*	3	PQ358843.1
15	*Fusarium warna*	1	MN954346.1
16	*Fusarium euwallaceae*	1	PP325881.1
17	*Fusarium solani*	13	NR163531.1
18	*Lasiodiplodia theobromae*	9	MZ089464.1
19	*Neoroussoella heveae*	1	OR760580.1
20	*Neoroussoella solani*	2	OQ704272.1
21	*Nigrograna chromolaenae*	2	MZ781246.1
22	*Nigrograna hydei*	1	ON911614.1
23	*Nigrograna mackinnonii*	4	MG063815.1
24	*Penicillium parvulum*	5	NR158790.1
25	*Penicillium citrinum*	14	OR764848.1
26	*Phanerodontia chrysosporium*	1	PP101334.1
27	*Purpureocillium lilacinum*	1	PV537465.1
28	*Trametes cubensis*	1	OL549154.1
29	*Trichoderma atroviride*	1	NR077207.1
30	*Xenoroussoella triseptata*	1	PV742932.1

### 3.2 Functional validation of heartwood formation-inducing fungi

#### 3.2.1 Fungal induction of heartwood formation in *D. odorifera*

**Figure 2:**
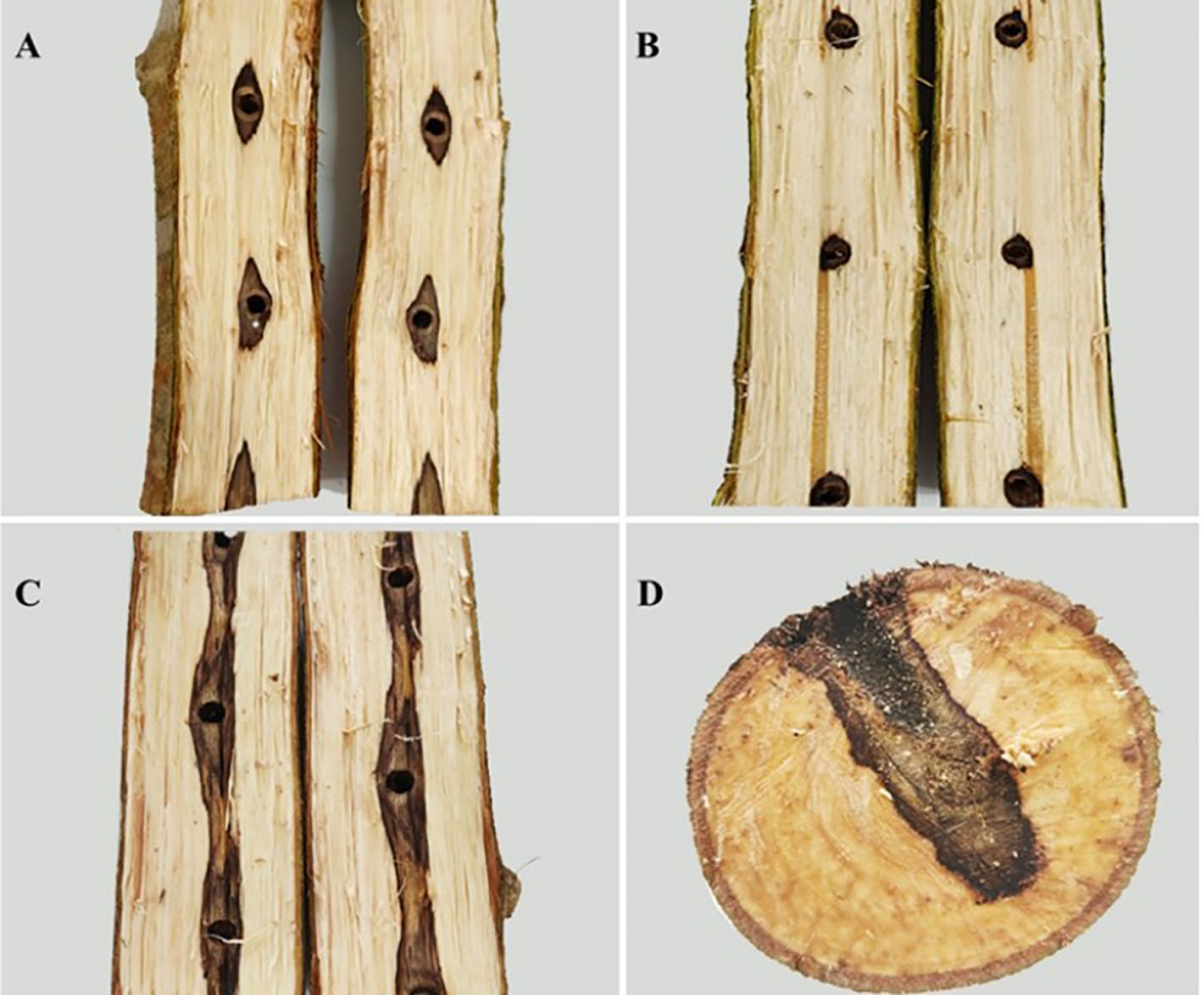
Induced heartwood formation samples in *D. odorifera.* **(A)** Inducing the Formation of Heartwood by Drilling(Ctrl-D); **(B)** Inducing the Formation of Heartwood by Drilling + PDB(Ctrl-DPDB); **(C)** Inducing the Formation of Heartwood by P3BM2 (Treat-DP); **(D)** Cross Section of Sample Induced by Treat-DP.

To verify the function of the purified fungi in inducing heartwood formation in *D. odorifera*, an induction experiment was conducted. Among them, strain separated from the sapwood named P3BM2 exhibited a prominent induction effect. Therefore, we selected P3BM2 as the representative strain for in-depth analysis of its induction mechanism, metabolic regulation effects, and related biological characteristics in the present manuscript. After a six-month induction period, heartwood formation was induced in *D. odorifera* across all treatments: Drilling + inoculation with fungus P3BM2 (Treat-DP), Drilling only (Ctrl-D), and Drilling + PDB medium (Ctrl-DPDB). The length of the discolored heartwood region in the P3BM2-induced samples was significantly greater than that in the control groups. The heartwood exhibited a darker color, and both its longitudinal extension and thickness were superior to those in the controls (**Figure 2**). Fungus P3BM2 demonstrated a significant induction effect, confirming its function in accelerating heartwood formation in *D. odorifera*. This strain can be developed into a fungal inducer for the production of *D. odorifera* heartwood.

#### 3.2.2 Morphological characteristics of fungal strain P3BM2

**Figure 3:**
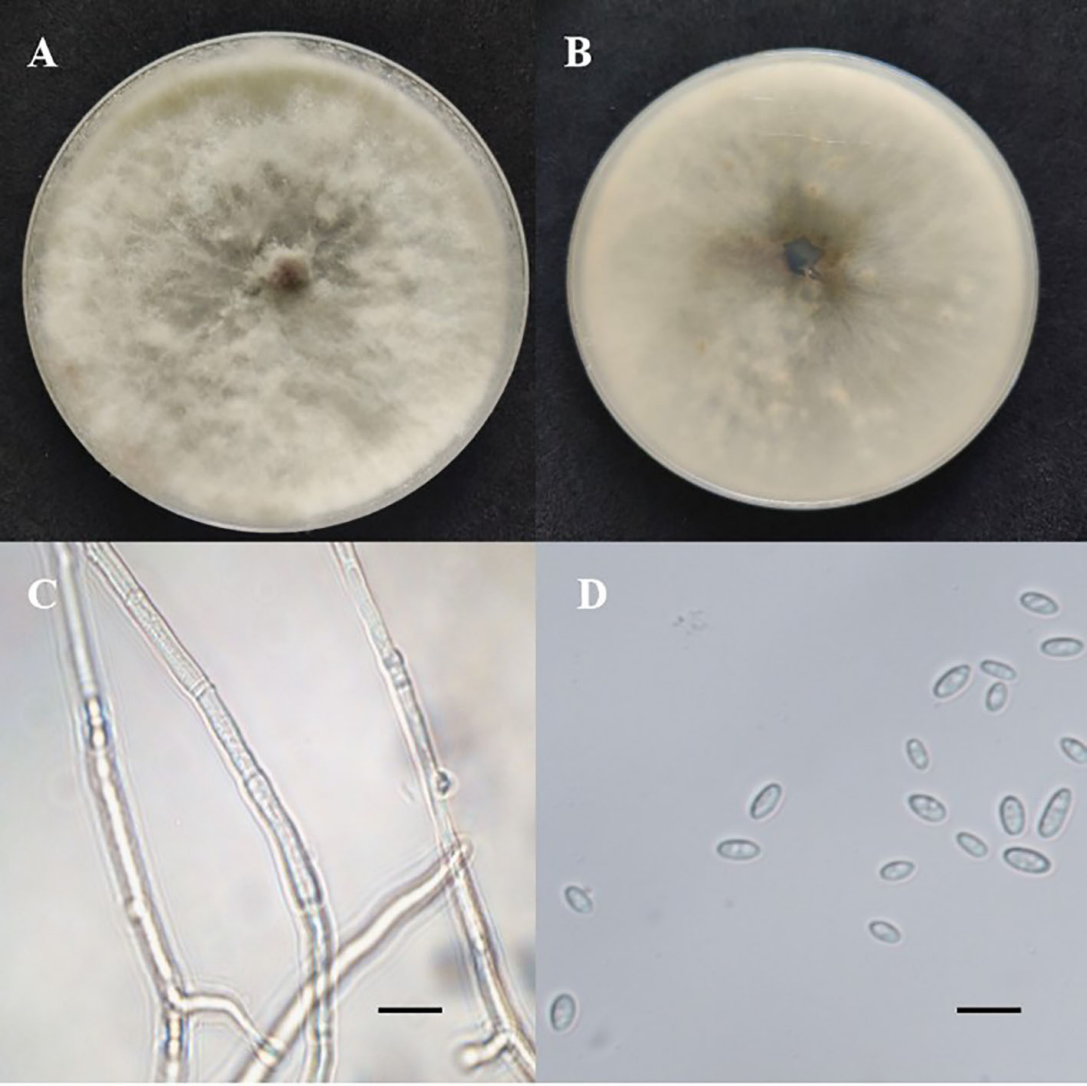
Morphological and microscopic identification of fungal strain P3BM2. **(A)** Frontal view of the fungal colony on the initial culture medium, **(B)** Reverse view of the fungal colony on the culture medium, **(C)** Microscopic observation of fungal mycelium, **(D)** Microscopic observation of fungal spores. Scale bar = 25 μm (applicable to panels C and D).

To further characterize the isolated fungi, morphological identification was performed on the strains used in the induction experiment. Strain P3BM2 grew rapidly on PDA medium at 28 °C, with a colony expansion rate reaching 5 cm per day. On average, it fully covered a 9 cm diameter Petri dish within 2 days. Initially, the colony appeared white or grayish-white with septate hyphae and dense, velvety aerial mycelium radiating outward (**Figure 3**). Streaked patterns were visible on the reverse side of the medium, spreading from the center to the periphery. After 3 days, the mycelium began developing an olive-green coloration. Over time, the color darkened from the center outward, transitioning to gray-brown and eventually turning black. Around 30 days, spherical pycnidia formed. The early fruiting bodies were gray-black, measuring 4–5 mm in diameter, and covered with velvety mycelium. Later, they became black and columnar, 3–4 mm in diameter and 10–20 mm in height. The immature conidia were elliptical, translucent, and unicellular, approximately 15 μm wide with a length-to-width ratio of 1:1.3–1.8. Mature spores darkened to brown and developed a single transverse septum.

#### 3.2.3 Molecular identification

**Figure 4:**
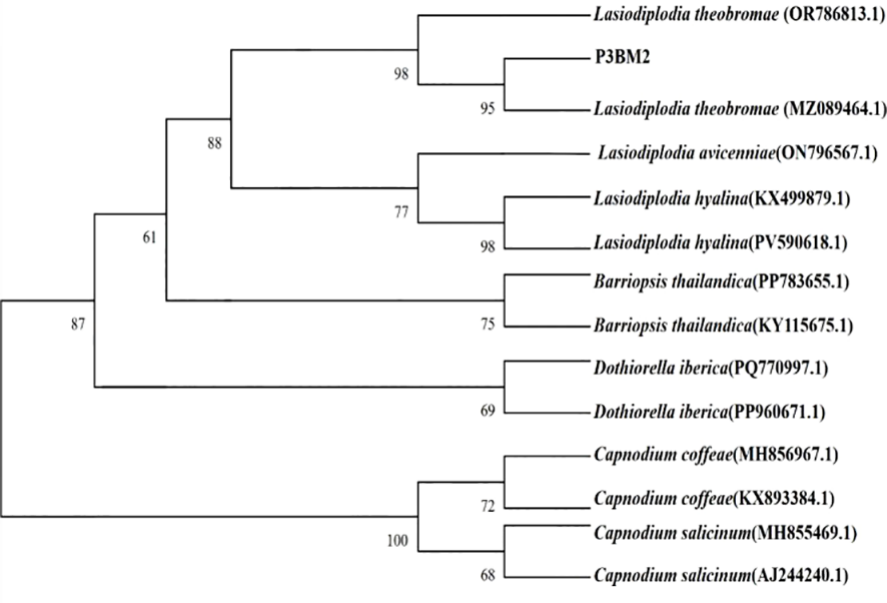
Phylogenetic tree of strain P3BM2 constructed by neighbor-joining method based on ITS sequences.

To identify the species of the inducing fungus, PCR amplification of the endophytic fungus P3BM2 from *D. odorifera* was performed using primers ITS1 and ITS4, yielding a target fragment of 516 bp. A phylogenetic tree was constructed using the Neighbor-Joining Method (**Figure 4**). Strains of different genera formed relatively independent clades. All *Lasiodiplodia* strains aggregated into a monophyletic clade with a bootstrap support value of 88. Within this clade, two *L. hyalina* reference strains formed a sub-branch with a high bootstrap support, reflecting a close intraspecific evolutionary relationship. P3BM2 clustered tightly within the *L. theobromae* clade, forming a sub-branch with *L. theobromae*(OR786813.1) and *L. theobromae*(MZ089464.1). The bootstrap support values for these branching relationships were 98 and 95. These high support values indicated that P3BM2 had a close evolutionary relationship with *L. theobromae*, supporting its classification as *L. theobromae*. It belonged to the fungal kingdom, *Ascomycota*, *Dothideomycetes*, *Botryosphaeriales*, *Botryosphaeriaceae*, *Botryosphaeria*, and was the asexual form of *Botryosphaeria rhodina*.

#### 3.2.4 Thin-layer chromatography identification

**Figure 5:**
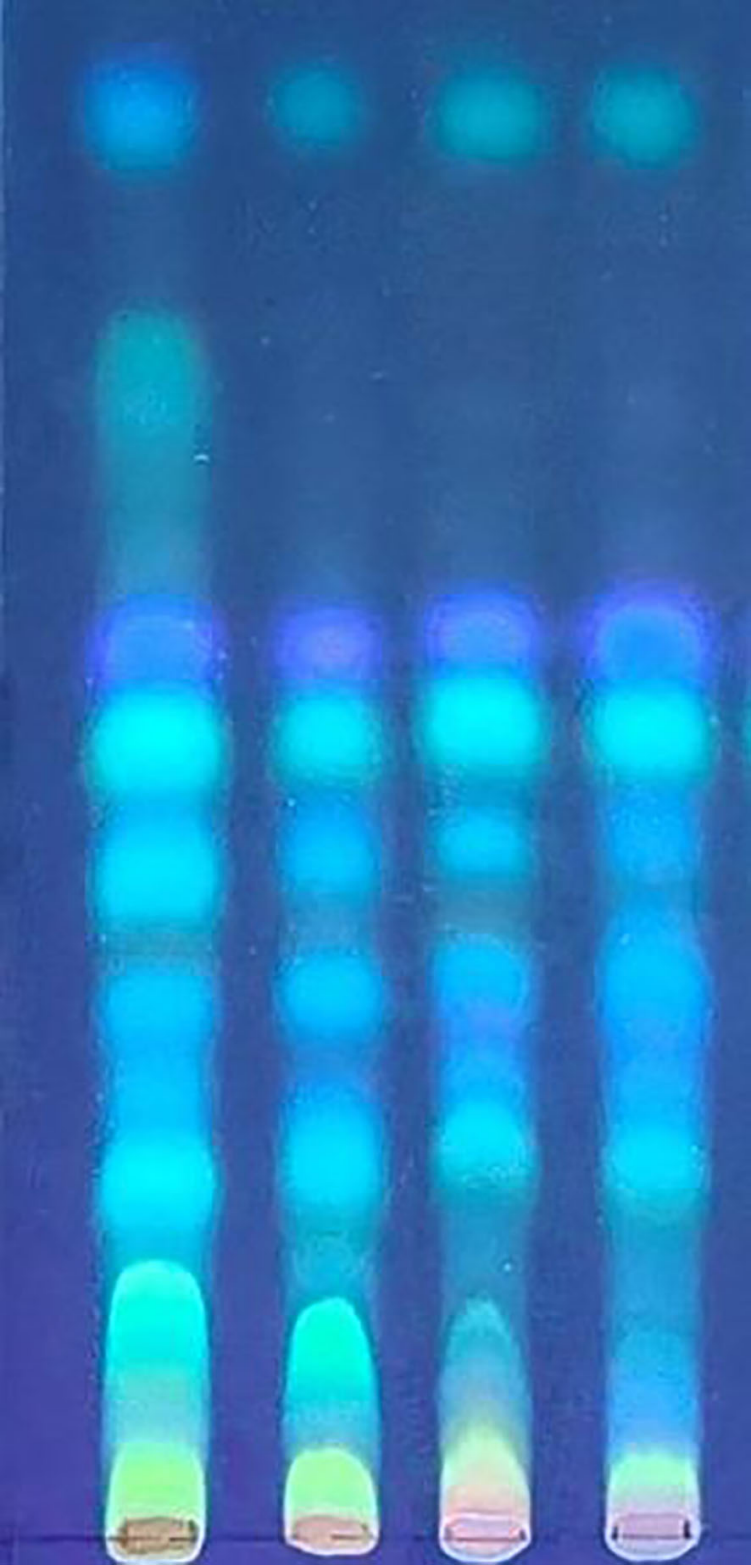
Thin-layer chromatography (TLC) identification results. From left to right: Ctrl-S (reference standard specified in the Chinese Pharmacopoeia), Ctrl-D (drilling-only sample), Ctrl-DPDB (sample with drilling and culture medium supplementation), and Treat-DP (fungal treatment group).

In accordance with the specifications of the Chinese Pharmacopoeia, the induced products of *D. odorifera* were identified using TLC. The thin-layer chromatography results from the heartwood induction experiment in *D. odorifera* were shown in **Figure 5**. Ctrl-S was the reference standard sample specified by the Chinese Pharmacopoeia, Ctrl-D was the sample with only drilling, and Ctrl-DPDB was the sample with drilling and added culture medium. In the chromatogram of the sample inoculated with the *L. theobromae* strain for six months, fluorescent spots corresponding in position and color to those of the reference standard material were observed, meeting the specifications of the Chinese Pharmacopoeia. The results indicated that *L. theobromae* could promote heartwood formation in *D. odorifera*, producing a substantial yield that complies with pharmacopoeial standards.

### 3.3 Metabolomic analysis

#### 3.3.1 Comparative metabolomics between Ctrl-D and treat-DP

**Figure 6:**
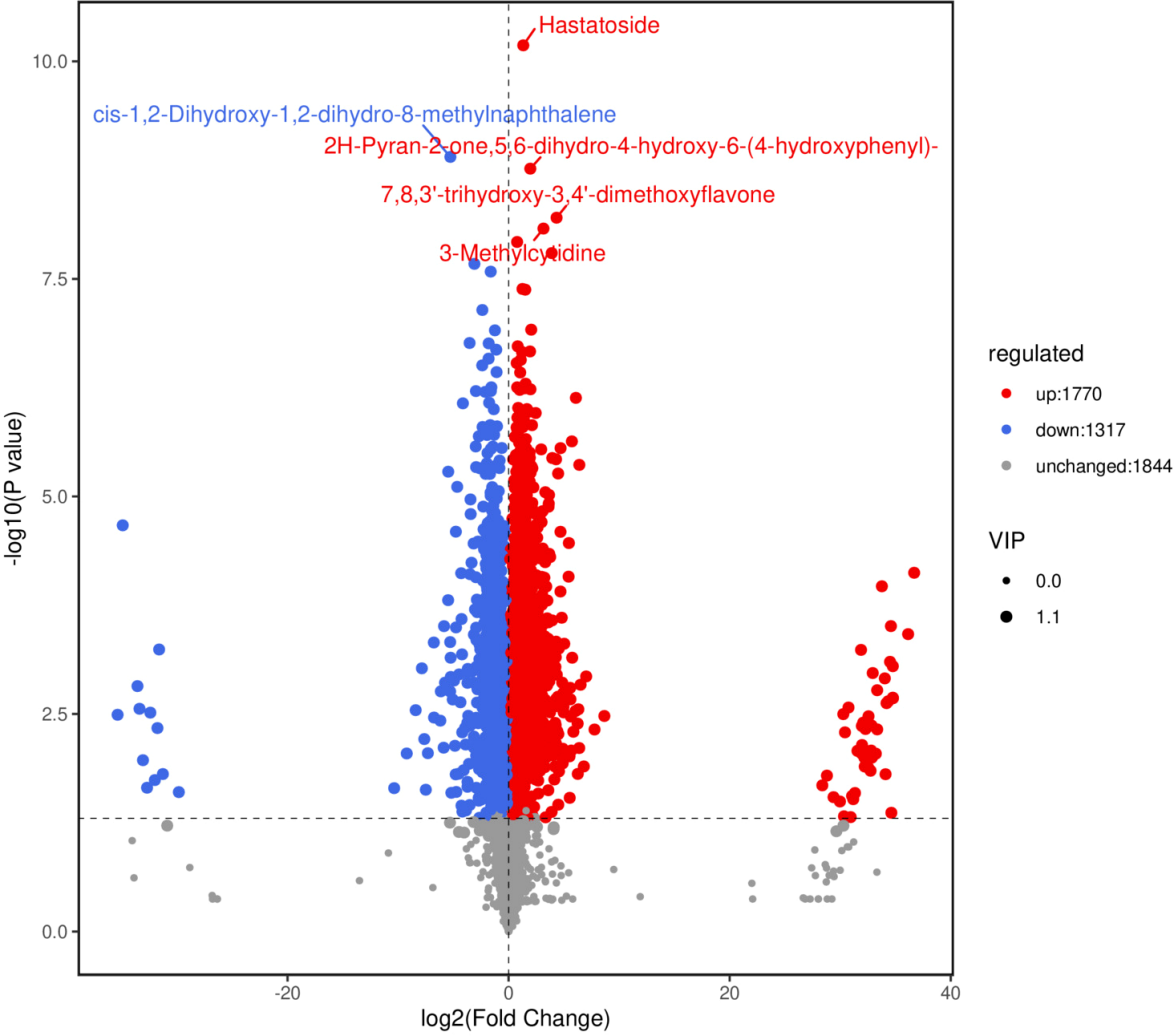
Volcano plot of differential metabolites in Ctrl-D -vs-Treat-DP comparison. Ctrl-D refers to the drilling-only sample, and Treat-DP is the fungal treatment group. Each dot represents one metabolite. The x-axis is log2 fold change (log2FC) of metabolites between groups. The y-axis is -log10 *P*-value from t-test. Dot size corresponds to VIP value of the OPLS-DA model (larger = more reliable differential metabolites). Blue dots: down-regulated metabolites; red dots: up-regulated metabolites; gray dots: non-significant metabolites. The top 5 identified metabolites (ranked by *P*-value) are labeled.

**Figure 7:**
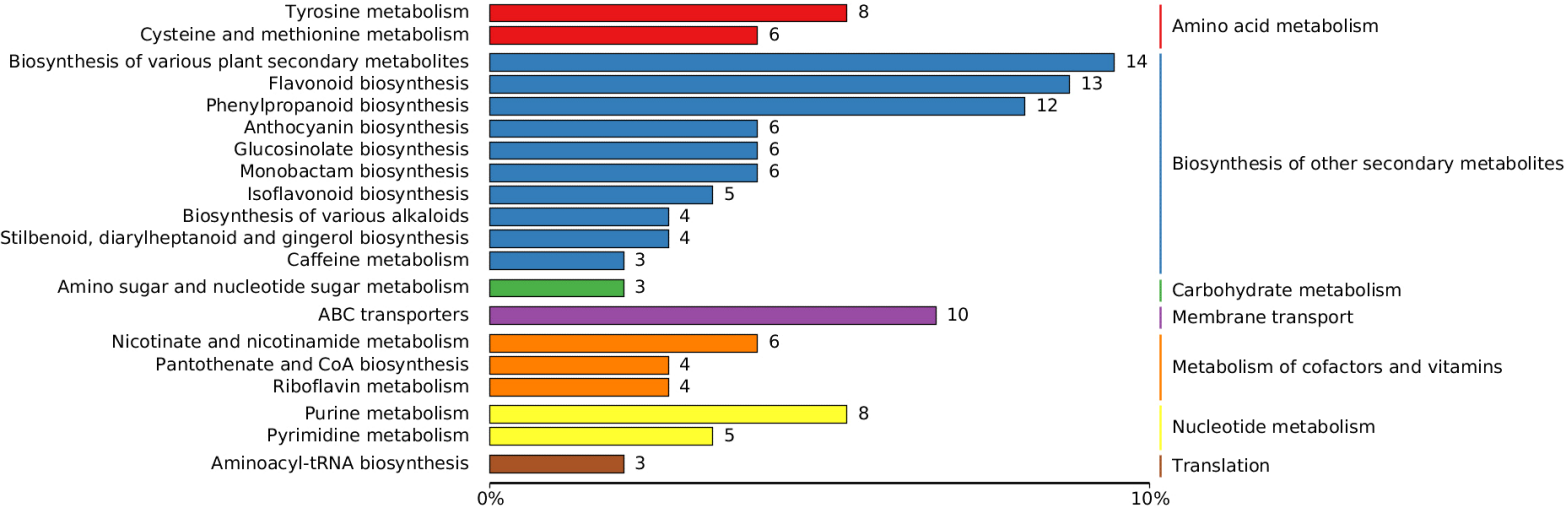
Pathway classification map of differential metabolites in Crtl-D -vs-Treat-DP comparison. Ctrl-D refers to the drilling-only sample, and Treat-DP is the fungal treatment group. Entries under the same box represent hierarchical classification annotations of KEGG pathways, corresponding to KO pathway level 2 and KEGG pathway names. The length of each bar indicates the number of differential metabolites annotated to the respective pathway.

**Figure 8:**
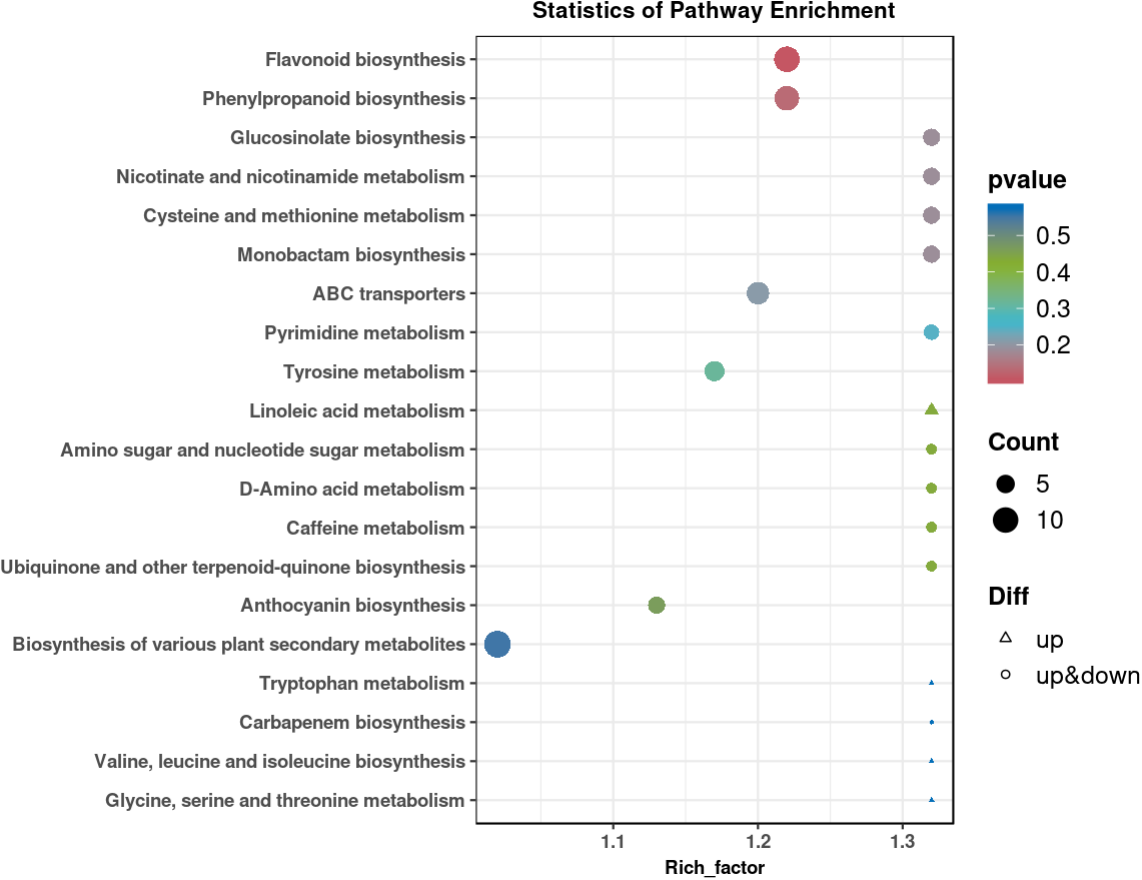
Analysis of KEGG enrichment and cumulative difference of metabolites in major metabolic pathways of Ctrl-D -vs-Treat-DP. Ctrl-D refers to the drilling-only sample, and Treat-DP is the fungal treatment group. Each dot represents one KEGG pathway. The x-axis is Rich Factor (ratio of differential-to-total metabolite proportion in the pathway); higher values indicate more significant enrichment. The y-axis shows pathway names. Dot color depth corresponds to the P value (smaller = more reliable significance). Dot size reflects the number of differential metabolites (larger = more metabolites). Dot shape denotes regulation: upward triangles (only up-regulated), downward triangles (only down-regulated), circles (both). Dots closer to the top-right corner have higher reference value.

Two control groups and one treatment group were set: the basic control group (drilling only) was denoted as Ctrl-D; the blank carrier control group (drilling + PDB) was denoted as Ctrl-DPDB; the treatment group (drilling + P3BM2) was denoted as Treat-DP. Metabolites from the control group Ctrl-D and the Treat-DP fungal treatment were analyzed, revealing a total of 4,931 metabolites. Among these, 1,770 were upregulated, 1,317 were downregulated, and 1,844 common metabolites showed no significant differences. It was found that the major differential metabolites belonged to iridoid glycosides, naphthalene derivatives, pyranone compounds, flavonoids, and alkaloids. With the exception of naphthalene derivatives, the contents of the other four compound classes were upregulated in the fungal-induced group (**Figure 6**). The upregulation of iridoid glycosides, pyranones, flavonoids, and alkaloids might, on one hand, enhance the stress resistance of *D. odorifera* as products of plant defense responses. On the other hand, these compounds contributed to the efficacy of the traditional medicine “Jiangxiang” involving anti-inflammatory, swelling reduction, pain relief, and blood circulation promotion (He, 2024). In the comparison between the drilling-only control group and the fungal treatment group, major differential metabolites were enriched in 130 metabolic pathways. Among these, the biosynthesis of various plant secondary metabolites, flavonoid biosynthesis, phenylpropanoid biosynthesis, tyrosine metabolism, and purine metabolism showed significantly enhanced activity in the fungal treatment group, accompanied by increased synthesis of related metabolites (**Figure 7**). The enhanced responses in flavonoid biosynthesis, phenylpropanoid biosynthesis, tyrosine metabolism, and purine metabolism revealed the intensified metabolic pathway activity in *D. odorifera* induced by the fungus. This not only strengthened the plant’s stress resistance but also directly promoted the synthesis and accumulation of key medicinal active components, as these pathways were central to the biosynthesis of critical medicinal compounds in *D. odorifera*. For Flavonoid biosynthesis and Phenylpropanoid biosynthesis, these two pathways exhibited extremely low *P*-values, a high Rich_factor, and relatively large node sizes (corresponding to a Count of 10). These features indicated that they were core pathways characterized by significant enrichment, a large number of differential metabolites, and high enrichment levels, and thus represented the key metabolic pathways for the Treat-DP relative to the Ctrl-D (**Figure 8**).

The observed changes in key metabolites provided important insights into potential metabolic regulatory mechanisms and offered a scientific basis for identifying potential biomarkers. These findings indicated that *L. theobromae* influenced the metabolism of *D. odorifera*, and these metabolic alterations might play a crucial role in the plant’s growth and heartwood formation.

#### 3.3.2 Metabolomic analysis of Crtl-DPDB vs. Treat-DP

**Figure 9:**
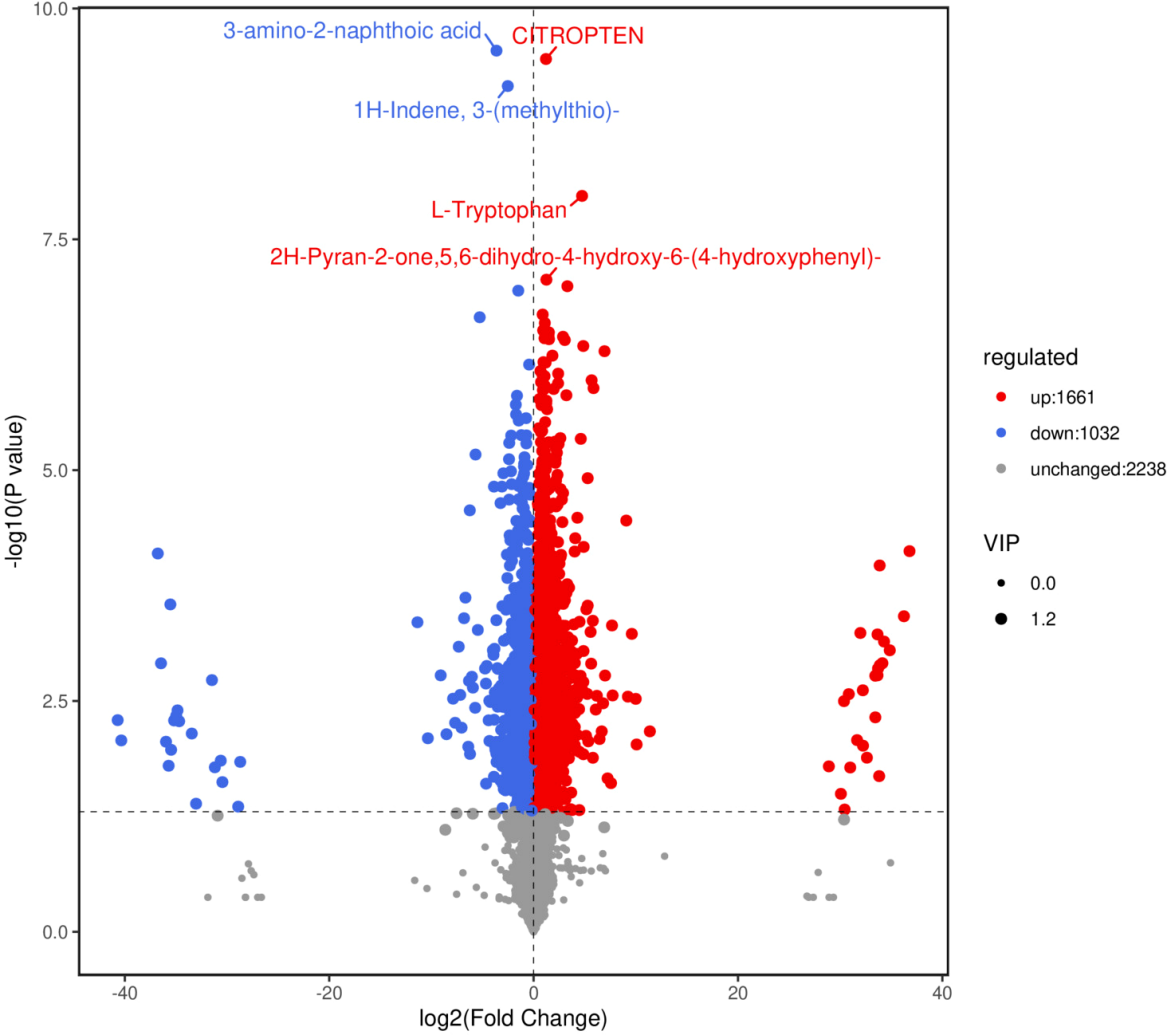
Volcano plot of differential metabolites in Ctrl-DPDB -vs-Treat-DP comparison. Ctrl-DPDB refers to the sample with drilling and culture medium supplementation, and Treat-DP is the fungal treatment group.

Metabolomic analysis was conducted comparing the control group Crtl-DPDB with the Treat-DP fungal treatment. A total of 2,693 differential metabolites were identified between the Crtl-DPDB control group and the fungal treatment group, enriched in 65 metabolic pathways. Among these, 1,661 metabolites were upregulated, 1,032 were downregulated, and 2,238 metabolites showed no significant differences(**Figure 9**). Flavonoids, organic acids, lipids, terpenoids, ketones, aldehydes, and esters constituted a large proportion of all upregulated differential metabolites. The top five identified metabolites belonged to aromatic compounds, coumarins, pyranones, and amino acids, with tryptophan and limonene being upregulated differential metabolites. The contents of aromatic compounds, coumarins, pyranones, and amino acids influenced heartwood color variation and affected the alcohol-soluble extractives and volatile oil content of the heartwood. Fungal induction altered the levels of these metabolites, thereby influencing the quality of the heartwood in *D. odorifera*.

**Figure 10:**
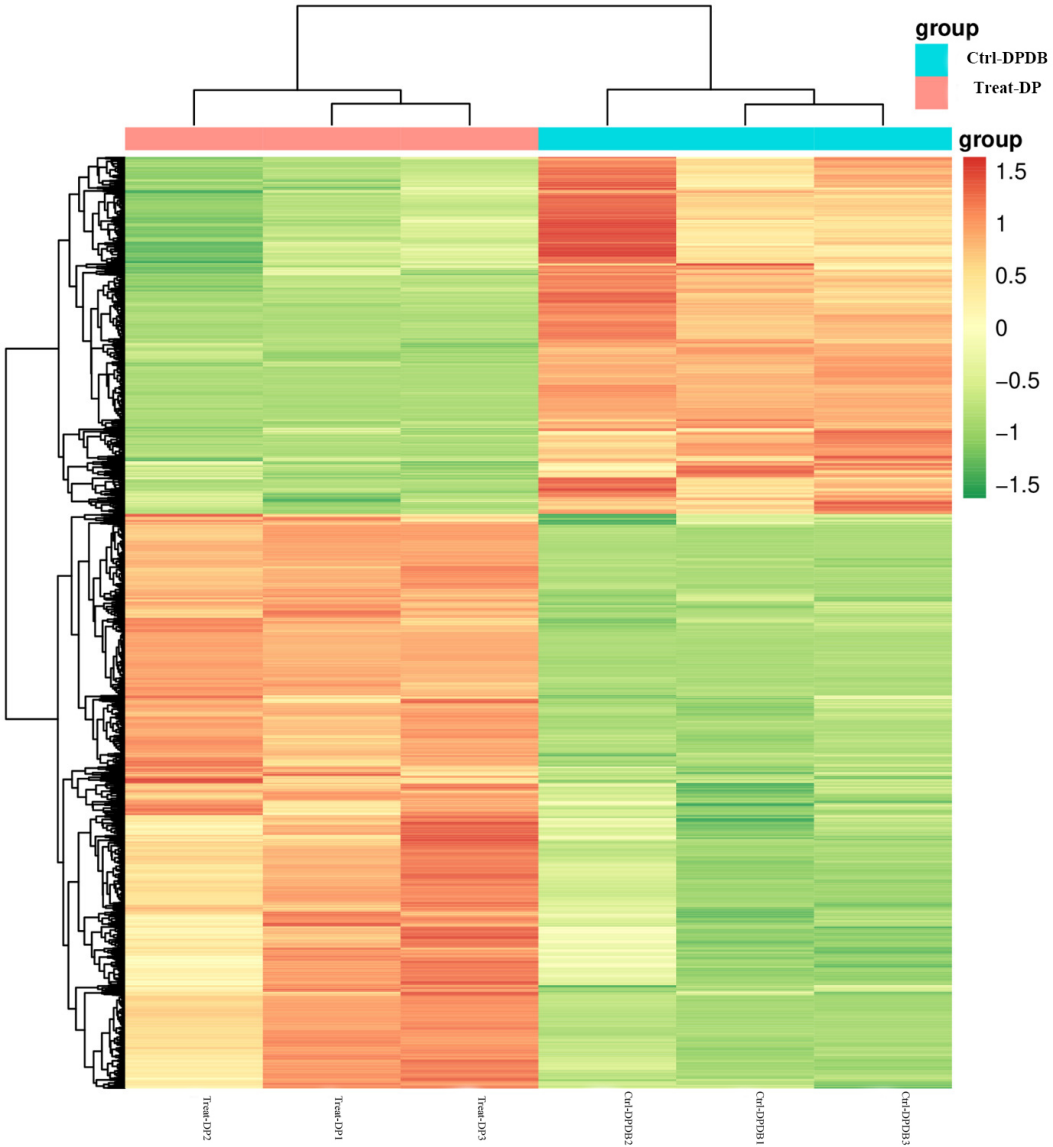
Cluster heatmap of differential metabolites in Ctrl-DPDB -vs-Treat-DP comparison. Ctrl-DPDB refers to the sample with drilling and culture medium supplementation, and Treat-DP is the fungal treatment group. The x-axis represents individual samples, while the y-axis denotes the Z-score normalized quantitative values of hierarchically clustered metabolites.

Compared to Crtl-DPDB, the highly expressed differential metabolites in the P3BM2 treatment group included amino acids, phenylpropanoids, alcohols, polyphenols, nucleotides, flavonoids, quinones, lignans, sugars, terpenoids, ketones/aldehydes/esters, vitamins, coumarins, organic acids, steroids, lipids, alkaloids, and others (**Figure 10**). Among these, ketones, aldehydes, and esters showed the highest proportion of upregulated substances, with 273 compounds accounting for 16.44%. Flavonoids followed, with 139 upregulated substances, representing 8.37%. The numbers of upregulated substances detected for lipids, organic acids, and terpenoids were 126 (7.59%), 117 (7.04%), and 100 (6.02%), respectively. **Table 3** presents the top 10 upregulated and top 10 downregulated metabolites with the largest fold changes in each group. It shows that compared with the Drilling group and PDB group, the content of metabolites such as flavonoids, iridoid glycosides, pyranones, carbohydrates, and amino acid derivatives in the fungus-treated group was upregulated. Flavonoids influenced heartwood color formation, while the high abundance of ketones, aldehydes, esters, and flavonoids indicated that during fungal-induced heartwood formation, the heartwood color gradually deepened, and medicinal compounds continuously accumulated.

**Table 3:** Main differential metabolites of Ctrl-D -vs-Treat-DP and Ctrl-DPDB -vs-Treat-DP.

Group	Main up-regulated differential metabolites	Classification of metabolites	Main down-regulated differential metabolites	Classification of metabolites
Ctrl-D -vs-Treat-DP	1-Methyl-4-hydroxy-6,8-dimethoxy-2-chinolon	Quinolones	5beta,9betaH,10alpha,3,4-seco-labd-7,13(Z)-dien-3,15 dioic acid	Diterpenoids
	3-(2-(4-hydroxyphenyl)-2-oxoethyl)-5,6-dihydropyridin-2(1H)-one	Pyridones	Yuanhuadine	Diterpenoids
	CMP-2-aminoethylphosphonate	Phosphonates	NGC0038531-01_C31H44O12_(2E)-3,7-Dimethyl-2,6-octadien-1-yl 3-O-(6-deoxy-alpha-L-mannopyranosyl)-4-O-[(2E)-3-(4-hydroxyphenyl)-2-propenoyl]-beta-D-glucopyranoside	Terpenoid Glycosides
	(2S)-1-((2S)-2-amino-3-methylbutanoyl)pyrrolidine-2-carboxylic acid	Amino Acid Derivatives	Leucylproline	Dipeptides
	(3S,4R)-3,4-Dihydroxycyclohexa-1,5-diene-1,4-dicarboxylate	Cyclohexadienes	(25R)-2alpha-hydroxy-5alpha-spirostan-3beta-yl O-beta-D-glucopyranosyl-(1->2)-O-[beta-D-glucopyranosyl-(1->3)]-O-beta-D-glucopyranosyl-(1->4)-beta-D-galactopyranoside	Steroidal Saponins
	HAEMATOMMIC ACID, ETHYL ESTER	Acid Esters	Acetamide, N-(1-methylethyl)-N-(4-methyl-2-oxazolyl)-	Acetamide Derivatives
	Cornuside	Iridoid Glycosides	1H-indol-2-yl-(1-methyl-3,6-dihydro-2H-pyridin-4-yl)methanone	Indole Pyridinones
	Glycine, 5-oxo-L-prolyl-	Amino Acid Derivatives	(all-Z)-5,7’-dihydroxy-2’-nonadeca-4,7,10,13,16-pentenyl-chromone	Chromones
	swertiabixanthone I 8’-O-beta-D-glucopyranoside	Xanthone Glycosides	2,5-Piperazinedione,3-(4-aminobutyl)-,(S)-(9CI)	Piperazinediones
	2-imino-3-methylene-5-L-(carboxy-L-valyl)pyrrolidine	Pyrrolidine Derivatives	Anabaenolysin B	Peptides
Ctrl-DPDB -vs-Treat-DP	1-Methyl-4-hydroxy-6,8-dimethoxy-2-chinolon	Quinolones	NGC0038531-01_C31H44O12_(2E)-3,7-Dimethyl-2,6-octadien-1-yl 3-O-(6-deoxy-alpha-L-mannopyranosyl)-4-O-[(2E)-3-(4-hydroxyphenyl)-2-propenoyl]-beta-D-glucopyranoside	Terpenoid Glycosides
	3-(2-(4-hydroxyphenyl)-2-oxoethyl)-5,6-dihydropyridin-2(1H)-one	Pyridones	Yuanhuadine	Diterpenoids
	(3S,4R)-3,4-Dihydroxycyclohexa-1,5-diene-1,4-dicarboxylate	Cyclohexadienes	(25R)-2alpha-hydroxy-5alpha-spirostan-3beta-yl O-beta-D-glucopyranosyl-(1->2)-O-[beta-D-glucopyranosyl-(1->3)]-O-beta-D-glucopyranosyl-(1->4)-beta-D-galactopyranoside	Steroidal Saponins
	9-Octadecenoic acid,12-hydroxy-, 2-(2-hydroxyethoxy)ethyl ester, [R-(Z)]- (9CI)	Fatty Acid Esters	2,5-Piperazinedione,3-(4-aminobutyl)-,(S)-(9CI)	Piperazinediones
	Streptomycin	Aminoglycosides	Acetamide, N-(1-methylethyl)-N-(4-methyl-2-oxazolyl)-	Acetamide Derivatives
	1H-Pyrano[3,4-c]pyridin-1-one,5-ethenyl-3,4-dihydro-4-(hydroxymethyl)- (9CI)	Pyranopyridones	4-Fluorothreonine	Amino Acid Analogs
	Diglykokoll	Glycerides	3-ethynylcyclohex-2-en-1-one	Ethynylcyclohexenones
	1-(O-alpha-D-glucopyranosyl)-3R,27R-octacosanediol	Glycosides	Leucylproline	Dipeptides
	3-Pyridineacetic acid,5-amino-2-carboxy-1,4,5,6-tetrahydro-4-oxo-, (-)-	Pyridine Acetic Acids	3,6-dimethyl-6,7,8,11-tetrahydro-5H-cyclodecafuran-4-one	Cyclodecafuranones
	Quercetin-3-rhamnosyl-arabinosid	Flavonoid Glycosides	1H-Indole-3-propanamide,.(dimethylamino)-N-[3-(1-methylethyl)-7-(2-methylpropyl)-5,8-dioxo-2-oxa-6,9-diazabicyclo[10.2.2]hexadeca-1(14),1,15,15-tetraen-4-yl]-(9CI)	Indole Amides

**Figure 11:**
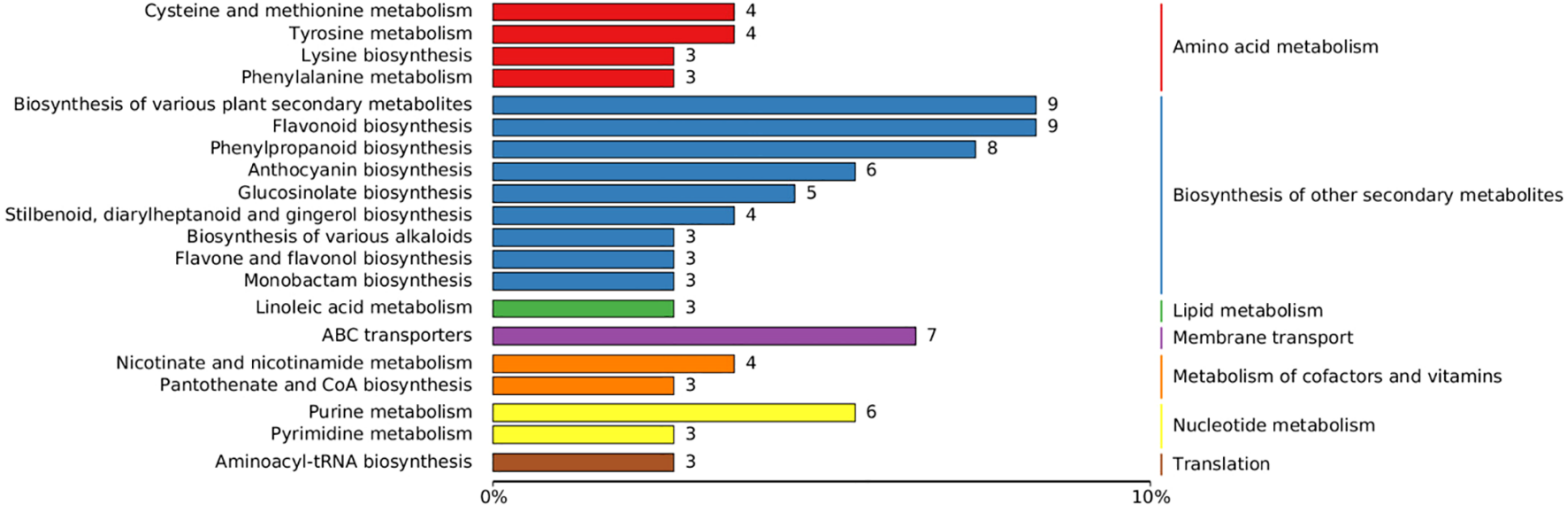
Pathway classification map of differential metabolites in Ctrl-DPDB -vs-Treat-DP comparison. Ctrl-DPDB refers to the sample with drilling and culture medium supplementation, and Treat-DP is the fungal treatment group.

**Figure 12:**
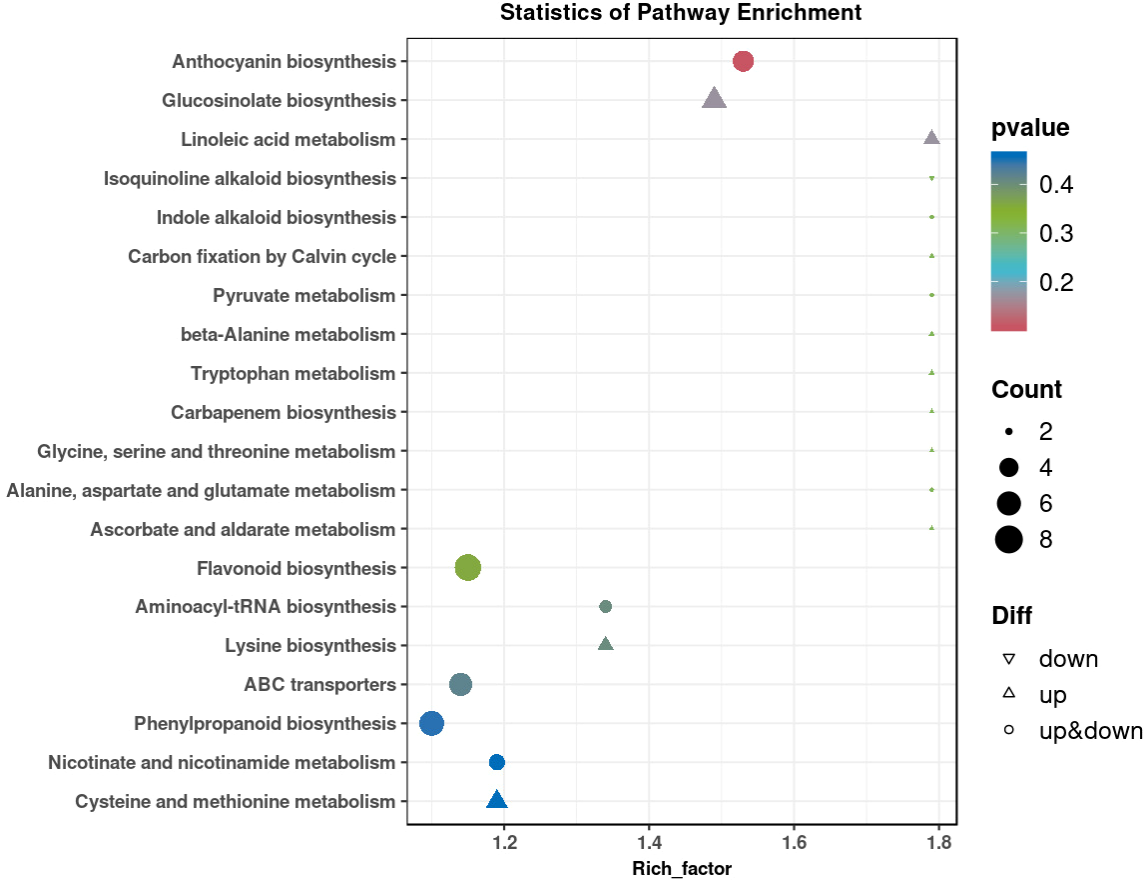
Analysis of KEGG enrichment and cumulative difference of metabolites in major metabolic pathways of Ctrl-DPDB -vs-Treat-DP. Ctrl-DPDB is sample with drilling and culture medium supplementation), Treat-DP is fungal treatment group.

The top 20 differential metabolic pathways involved 7 physiological functions and 93 differential metabolites, including amino acid metabolism, biosynthesis of other secondary metabolites, lipid metabolism, membrane transport, cofactor and vitamin metabolism, nucleotide metabolism, and translation (**Figure 11**). Three metabolic pathways in the *L. theobromae*-induced samples showed high differential significance (**Figure 12**), collectively enriching 14 categories of differential metabolites related to flavonoids, sugars, organic acids, amino acids, and lipids. These pathways were: 1. Anthocyanin biosynthesis, with flavonoids as the main differential metabolites; 2. Glucosinolate biosynthesis, with sugars, organic acids, and amino acids as the main differential metabolites; 3. Linoleic acid metabolism, with lipids as the main differential metabolites. All three pathways were closely associated with plant defense responses, serving as mechanisms for plants to cope with stress. This indicated that fungal treatment triggered a more intense defense response compared to the control group, thereby promoting heartwood formation.

## 4 Discussion

Plant endophytes are diverse, widely distributed, and can enhance plant stress resistance, insect resistance and produce beneficial secondary metabolites (Taulé et al., 2021). However, due to limitations in microbial isolation techniques, only about 1% of the natural microbial community can be successfully isolated and cultured artificially (Pan et al., 2025). Using the traditional tissue separation method, we identified endophytic fungi belonging to 2 phyla and 15 genera. The tissue separation method only captures cultivable strains, whereas a large proportion of fungi in *D. odorifera* are unculturable under artificial conditions. It should be noted that the tissue separation method only captures cultivable strains, and the present study focused on the screening and functional verification of single culturable strains in *D. odorifera*. In our isolates, the number of fungal strains isolated from the heartwood layer was higher than that from the sapwood layer, providing candidate strains for the artificial induction of heartwood formation in *D. odorifera*. Heartwood-specific strains may play a crucial role in promoting sapwood-to-heartwood transformation, which provides more options for the strain types employed in future artificial induction of heartwood formation.

A total of 30 species of endophytic fungal strains were isolated from the heartwood and sapwood of D. odorifera in this study, and all 30 species fungi were subjected to heartwood induction experiments following the same protocol. Among them, strain P3BM2 (*L. theobromae*) exhibited the most prominent induction effect, with the highest efficiency in promoting sapwood-to-heartwood transformation and significant accumulation of characteristic active components in the induced heartwood. Therefore, P3BM2 was selected as the representative strain for in-depth analysis of its induction mechanism, metabolic regulation effects, and related biological characteristics in the present manuscript. In this study, confirmed that *L. theobromae* has the function of promoting heartwood formation in *D. odorifera*. Additionally, the fungal treatment activated multiple metabolic pathways in *D. odorifera*, leading to the production of various differential metabolites. Compared to the drill-only control group (Ctrl-D), the contents of iridoid glycosides, pyranones, flavonoids, and alkaloids were significantly upregulated in the fungal-induced group. Several biosynthetic pathways, including flavonoid biosynthesis, phenylpropanoid biosynthesis, tyrosine metabolism, and purine metabolism, showed significantly enhanced activity in the fungal treatment group, accompanied by increased synthesis of related metabolites. Compared to the drill + PDB control group, the heartwood induced by *L. theobromae* exhibited three significantly different metabolic pathways, with upregulated differential metabolites primarily consisting of flavonoids, organic acids, lipids, terpenoids, ketones, aldehydes, and esters. The high expression of these metabolic pathways and the accumulation of these metabolites are closely associated with heartwood formation in *D. odorifera*. The metabolomics analysis identified differential metabolites between the P3BM2-inoculated group and the control group, which were significantly enriched in the phenylpropanoid biosynthesis pathway and flavonoid biosynthesis pathway. Phenylpropanoids are key precursors for lignin and phenolic compound synthesis—lignin deposition in cell walls is a typical anatomical feature of heartwood, while phenolic compounds are the main active components of *D. odorifera* heartwood. The upregulation of these pathways and metabolites suggests that P3BM2 may induce heartwood formation by activating the host’s secondary metabolism: on the one hand, promoting lignin synthesis to induce cell wall thickening and sapwood lignification; on the other hand, enhancing flavonoid and phenolic compound accumulation to form the characteristic chemical composition of heartwood.

Research indicates that heartwood formation in *D. odorifera* typically begins at 6–7 years of age, with heartwood diameter, heartwood area percentage, and heartwood-to-diameter ratio showing a highly significant positive correlation with tree age (Chen et al., 2016). Theoretically, artificial interventions such as pruning and external stimulation can promote heartwood formation in *D. odorifera* (Gartner et al., 2003). Some studies have employed methods like water stress, growth regulator injections, and fungal suspension infusion to alter the tree’s internal metabolism or directly accelerate the conversion of sapwood into heartwood (Jia, 2014). Several fungal species have been reported to induce heartwood formation in *D. odorifera* within 9–12 months. However, due to the limited understanding of their mechanisms of action and biological characteristics, their application in heartwood production remains challenging. The biological traits of fungal strains vary across species and genera, and even strains of the same species from different biological sources may exhibit distinct characteristics (Rana et al., 2020). To better apply fungal strains in *D. odorifera* heartwood production, further investigation into the biological properties and molecular mechanisms of heartwood-inducing fungi is necessary.

This study confirms that *L. theobromae* can promote heartwood formation in *D. odorifera* is widely distributed in tropical and subtropical regions and can cause diseases in various plants. Its biological characteristics vary depending on the host source. Diseases caused by *L. theobromae* are often associated with climatic conditions, physical wounds, plant vigor, and cultivar susceptibility (Jiang et al., 2025). It has been reported to cause leaf spot in oil tea (Tang et al., 2021), leaf spot in pineapple (Tang et al., 2021), leaf spot in sisal (Guan et al., 2022), and fruit rot in mango (Tang et al., 2016), as well as stem rot, leaf blight, and fruit rot in cacao (Huda-Shakirah et al., 2022)*. L. theobromae* itself produces secondary metabolites in response to various biotic and abiotic stimuli (Salvatore et al., 2020), including compounds such as cyclohexenes and cyclohexenones, indoles, jasmonates, lactones, melleins, and phenolics, which exhibit phytotoxic, cytotoxic, and antimicrobial activities. It has also been reported that *L. theobromae* can promote agarwood formation in *Aquilaria sinensis*. The functional outcomes of fungal infection may depend on the depth and duration of fungal invasion. Investigating the interaction mechanisms between plants and endophytes will help us selectively harness fungal functions for specific purposes. When *L. theobromae* invades avocado branches, it induces oxidative stress in the host, leading to cell necrosis and causing stem canker (Liu et al., 2022). Interestingly, oxidative stress is also a key factor promoting the transformation of sapwood into heartwood (Daymi et al., 2016). Although no disease symptoms were observed in our short-term induction experiment, *L. theobromae* is a known wood-rot pathogen in some contexts, long-term virulence and host-microbe interaction dynamics remain to be clarified. Additionally, wood-degrading enzymes are closely involved in lignin modification and cell wall remodeling, which are core physiological processes during sapwood-to-heartwood transformation. Evaluating the activity of these enzymes in *L. theobromae* and their expression patterns in inoculated host tissues will help reveal how the fungus modulates wood structure to promote heartwood formation. A deeper understanding of the biological characteristics of fungi and the molecular mechanisms underlying physiological responses will facilitate the stable application of fungi in production. In the future, high-throughput sequencing can be used to analyze and identify the types and abundance of microorganisms within trees, enabling the selection of suitable microbial strains for artificial induction of heartwood formation through methods such as microbial suspension injection (Li et al., 2025). Future research could further explore the principles of fungal-induced heartwood formation in *D. odorifera* using integrated multi-omics technologies.

Fungal community interactions are critical for natural heartwood formation. Due to the complexity of community interactions, this study initially focused on the function of single strains. In future studies, researchers can construct synthetic fungal communities with dominant strains from heartwood, evaluate the synergistic or competitive effects of multi-strain co-inoculation on heartwood induction, and combine high-throughput sequencing to analyze changes in the native fungal community during induction, which will help clarify the role of community interactions in heartwood formation. Future studies will focus on quantifying the time-course of heartwood development to clarify key induction dynamics, thereby fully elucidating the sequential process of fungal-induced heartwood formation in *D. odorifera*.

## Data Availability

The original contributions presented in the study are included in the article/supplementary material. Further inquiries can be directed to the corresponding authors.
